# An optimized mouse model of *Staphylococcus aureus* infected diabetic ulcers

**DOI:** 10.1186/s13104-022-06170-5

**Published:** 2022-09-07

**Authors:** Ana Isabel MENDES, Maria João PEIXOTO, Alexandra Pinto MARQUES, Jorge PEDROSA, Alexandra Gabriel FRAGA

**Affiliations:** 1 School of Medicine, Life and Health Sciences Research Institute (ICVS), University of Minho, Campus de Gualtar, 4710-057 Braga, Portugal; 2 ICVS/3B’s–PT Government Associate Laboratory, Braga, Guimarães, Portugal; 3 3B’s Research Group, I3Bs – Research Institute on Biomaterials, Biodegradables and Biomimetics, Headquarters of the European Institute of Excellence On Tissue Engineering and Regenerative Medicine, University of Minho, AvePark, Zona Industrial da Gandra, 4805-017 Barco, Guimarães, Portugal

**Keywords:** Chronic wounds, Diabetic mouse model, Impaired wound healing, Inflammation, MRSA infection

## Abstract

**Objective:**

Diabetic foot infection (DFI) represents a major healthcare burden, for which treatment is challenging owing to the pathophysiological alterations intrinsic to diabetes and the alarming increase of antimicrobial resistance. Novel therapies targeting DFI are therefore a pressing research need for which proper models of disease are required.

**Results:**

Here, we present an optimized diabetic mouse model of methicillin-resistant *Staphylococcus aureus* (MRSA)-infected wounds, that resemble key features of DFI, such as pathogen invasion through wound bed and surrounding tissue, necrosis, persistent inflammation and impaired wound healing. Thus, in a time-efficient manner and using simple techniques, this model represents a suitable approach for studying emerging therapies targeting DFI caused by MRSA.

**Supplementary Information:**

The online version contains supplementary material available at 10.1186/s13104-022-06170-5.

## Introduction

Management of chronic ulcerative wounds is a critical worldwide healthcare challenge, associated with a high risk of morbidity and mortality [1, 2]. Diabetes is an important predisposition factor for skin ulceration, particularly on the foot, which is often complicated by infection [3, 4]. Several pathogens can be found in diabetic foot infection (DFI), but *Staphylococcus aureus* is the most common [5–7]. *S. aureus* typically forms biofilms, evading the activity of both the host immune system and antibiotics, hampering current treatment strategies [6, 8–10]. The lack of effective treatment is further aggravated, when considering the alarming increase of methicillin-resistant *S. aureus* (MRSA) prevalence in DFI [7, 8]. Thus, the development of new therapeutic strategies for DFI is critical, for which appropriate and reliable models are urgently required. In this regard, this study developed an optimized protocol for generating standardized MRSA-infected wounds in a diabetic mouse model of impaired healing that mimics the main hallmark features of DFI, including continuous necrosis and inflammation associated with invasive infection of the wound bed, and can be easily employed to test novel therapies targeting DFI.

## Main text

### Methods

Animal experimentation was performed at the Life and Health Sciences Research Institute at the University of Minho, in accordance with the Directive 2010/63/EU, and approved by Institutional Animal Care and Use Committee of University of Minho. 8–12-week-old male C57BL/6 mice (Charles River Laboratories) were housed under specific pathogen-free condition with food and water ad libitum and acclimatized for 1 week before the experiment. Mice (n = 14) were equally and randomly divided in two groups corresponding to established endpoints of 2- and 9-days post-infection (dpi). Randomization was performed using randomize function of Microsoft^®^ Excel^®^. Humane endpoints were followed as described on Additional file 1: Table S1.

#### Diabetes induction

Type 1 diabetes mellitus (T1DM) was chemically induced, as previously described [11], administering 50 mg/kg of streptozotocin (STZ) (Merck KGaA, Germany) for 5 consecutive days. Blood glucose levels were measured 9 days after STZ treatment, using a monitor glucose device. Levels of blood glucose higher than 150 mg/dL were considered hyperglycemic. Mice were observed for signs of polydipsia and polyuria throughout the experimental period.

#### Dorsal fur depilation

On the day before surgery, dorsal fur of mice was shaved, using a hair clipper followed by depilatory cream for 1 min. Cream was further removed by wiping the skin with cotton soaked in warm water.

#### Inoculation of polycarbonate membranes

Briefly, 0.2 µm pore size polycarbonate membranes (Merck KGaA, Germany) were cut in 5-mm diameter discs, sterilized on both sides by UV light for 30 min and then placed on mannitol salt agar (MSA). A bacterial suspension of 10^8^ colony forming units (CFU)/ml of *S. aureus* Rosenbach (ATCC BAA 2313) was prepared using isolated colonies previously grown on MSA, that were resuspended in saline and further inoculated on the membranes (10^2^ CFU/membrane). Inoculated membranes were incubated overnight at 37 ºC to grow a biofilm (attaining approximately 10^9^ CFU/membrane).

#### Excisional wounding and infection

**Fig. 1:**
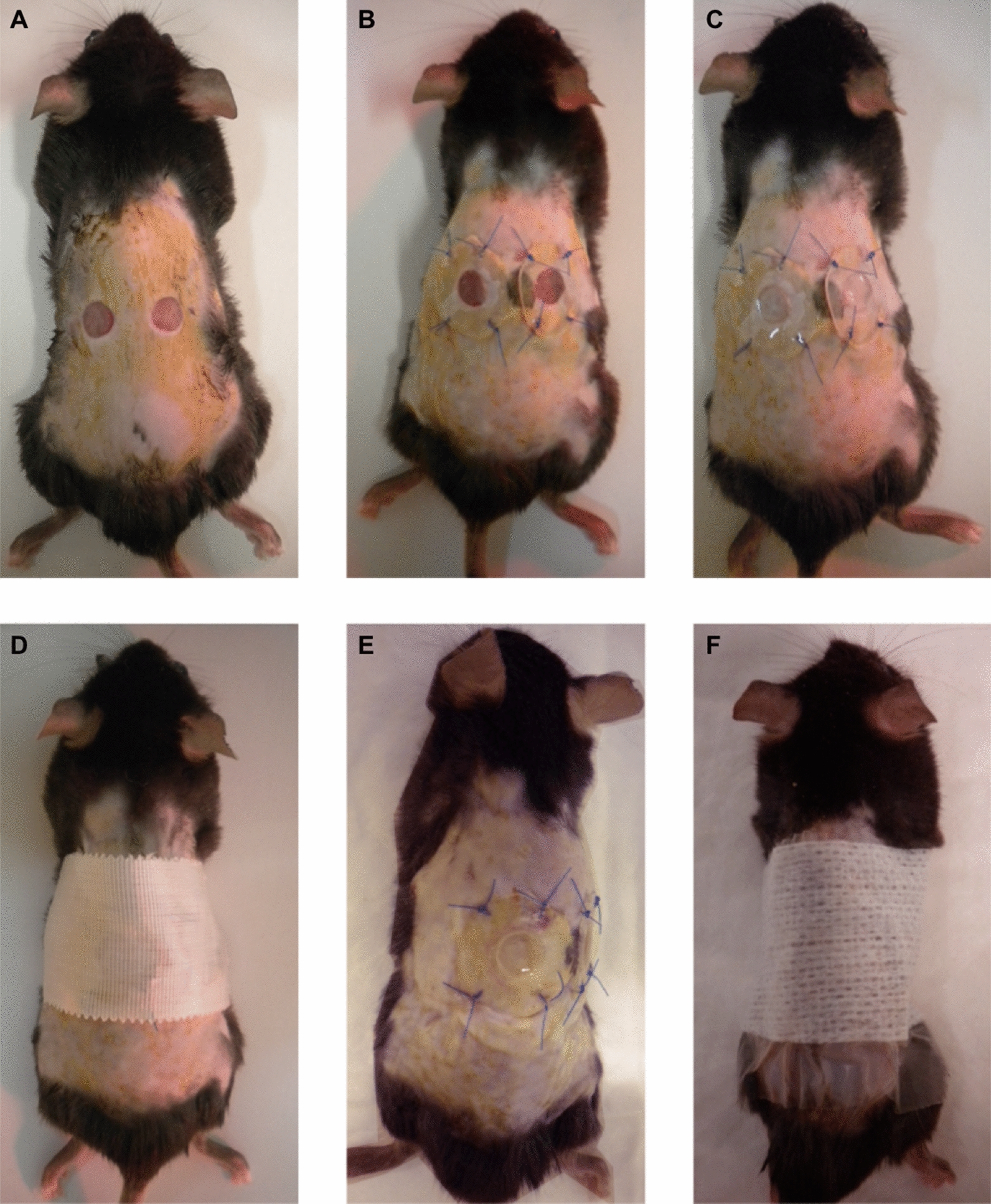
Illustration of the protocol of full-thickness excisional wounding, infection and dressing: **A** creation of two symmetrical full-thickness wounds using a punch biopsy; **B** splinted wounds with silicone rings secured with cyanoacrylate glue and four interrupted sutures; **C** wound’s infection with *S. aureus*-inoculated polycarbonate membranes; **D** wound’s covering with self-adhering bandage; **E** representative photo of biofilm covering the wound 2 dpi; **F** wound dressing with a sterile transparent semi-occlusive dressing followed by an elastic bandage, after polycarbonate membranes removal

Mice were intraperitoneally injected with anesthetics (75 mg/kg ketamine and 1 mg/kg medetomidine) and analgesic (0.1 mg/kg buprenorphine). Two symmetrical full-thickness excisional wounds were created using a 5-mm diameter punch biopsy, by placing mice on their side, pulling the dorsal skin and perforating through the folded skin (Fig. 1A). To minimize wound contraction, a silicone splint ring (15-mm external and 6-mm internal diameter) was positioned around each wound using cyanoacrylate glue and secured with four interrupted sutures of 5/0 nylon (Fig. 1B). During the procedure, wounds were maintained hydrated with saline.

Wounds were infected by placing the *S. aureus*-inoculated polycarbonate membrane face down for direct contact of biofilm with the wound bed (Fig. 1C). Finally, wounds were covered with Durapore™ self-adhering bandage (3 M, USA) (Fig. 1D). Mice received 1 mg/kg atipamezole intramuscularly to revert the effect of anesthesia and were then placed under a warming lamp until full recovery. For postoperative pain relief, analgesia was administered subcutaneously with maximal intervals of 12 h during the two following days. Mice also received a vitamin supplementation (Duphalyte^®^) by subcutaneous injection, to avoid massive weight loss and dehydration.

Two days after wounding/infection, a biofilm covering the wounds was observed (Fig. 1E). At this point, mice received a light sedative (7.5 mg/kg ketamine and 1 mg/kg medetomidine, intraperitoneally) to remove the polycarbonate membranes. Topical treatment can be applied at this point, if desired. A sterile transparent semi-occlusive dressing Tegaderm (3 M, USA) was then applied covering wounds and splints, followed by an Omnifix elastic bandage (Hartmann) (Fig. 1F).

#### Wound tissue analysis

To assess the validity of the proposed model, wound tissue was collected for microbiological and histological analysis at 2- and 9-dpi. Mice were sacrificed with an overdose of isoflurane, and dressings and splints were carefully removed. Using a scalpel blade, wounds and surrounding skin tissue were harvested. One wound was used for bacterial burden quantification, while the other was dissected for histological analysis. For bacterial quantification, wound tissue was minced, tenfold serially diluted in sterile saline and cultured on MSA at 37 ºC/24 h. The number of viable bacteria was expressed as Log_10_CFU/wound. For histological analysis, tissue was divided across the wound center and immersed in neutral buffered formaldehyde (4%, w/v), embedded in paraffin, and then sectioned in samples of 4-µm thickness for staining with Hematoxylin and Eosin (H&E) and Gram.

#### Statistical analysis

Data were reported as mean ± standard deviation. Differences were assessed through Student’s t-test, using Graphpad Prism 7 (Graphpad Software Inc., CA). Statistical significance was set at *p* < 0.05.

## Results

**Fig. 2:**
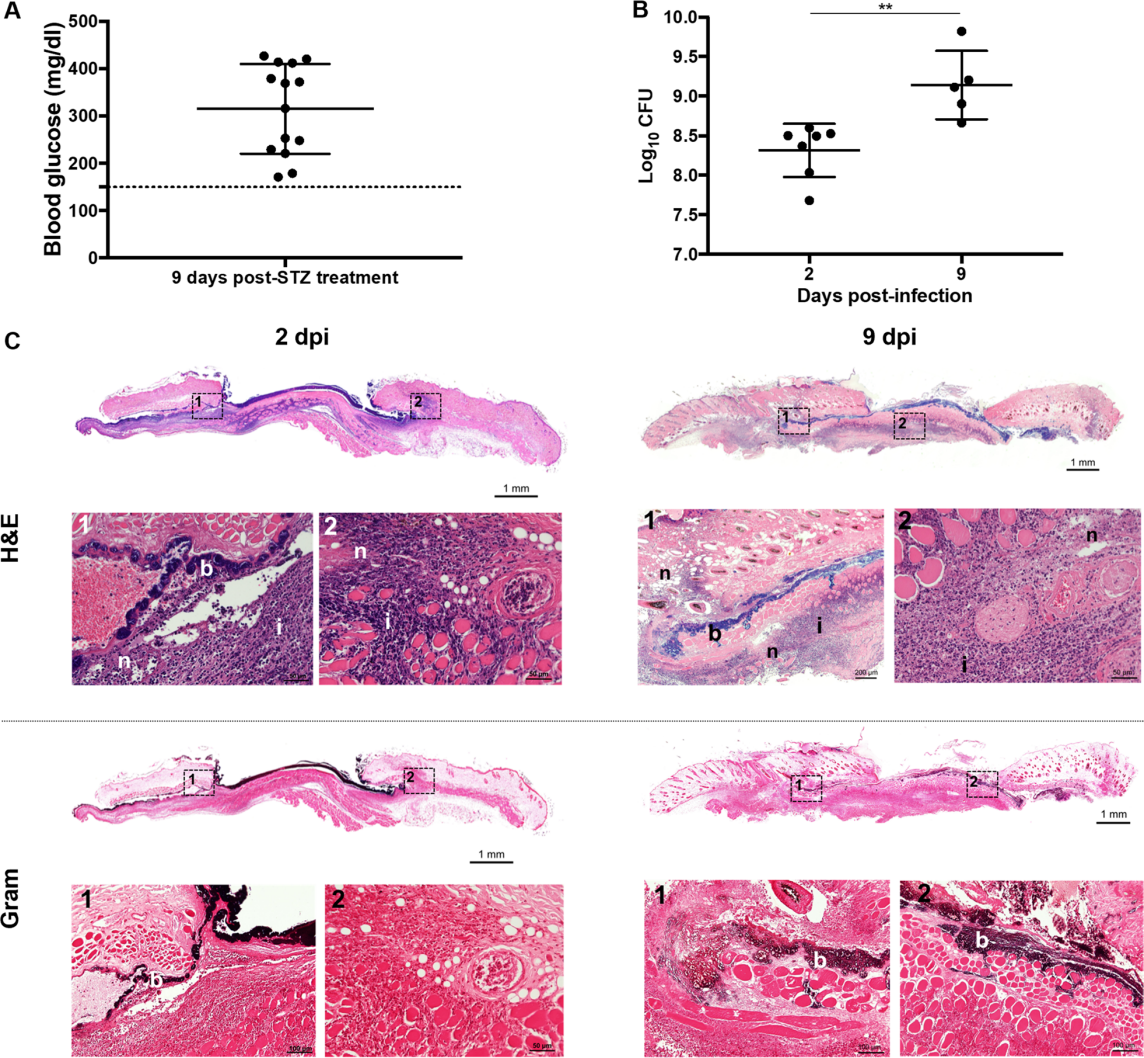
Representative outcomes of the protocol of MRSA-infected wounds in a diabetic mouse model: **A** Blood glucose levels (mg/dL) after 9 days of STZ treatment; **B** bacterial burden of wounds at 2- and 9-dpi; **C** H&E and Gram-stained sections of wounds at 2- and 9-dpi (b: bacteria; i: inflammatory immune cells; n: necrosis)

The implementation of diabetes was achieved with STZ by damaging insulin producing β cells of pancreatic islets. All animals were considered diabetic, showing blood glucose levels of 312.4 ± 90.8 mg/dL (Fig. 2A), excessive water consumption and urine production.

Regarding the infection of inflicted wounds, a biofilm covering the wound bed was clearly identified both macroscopically (Fig. 1E) and microscopically (Fig. 2C). The biofilm formation was identical in both wounds and among animals. At 2-dpi, wounds bacterial burden reached a mean log_10_CFU of 8.32 that significantly increased to 9.14 at 9-dpi (Fig. 2B). Histological analysis revealed that MRSA was present not only on the wound surface but was also able to spread into the surrounding non-wounded deep tissue. Furthermore, infection led to necrosis and instigated the infiltration of inflammatory cells in the vicinity of the areas of bacterial accumulation (Fig. 2C). Wounds remained open over the experimental period, without signs of fibroplasia or granulation tissue formation, and revealed an identical pattern of inflammation and infection along different sections of wound area. It is noteworthy that two animals from the 9-dpi experimental group succumbed before the established endpoint.

## Discussion

This study reports an optimized protocol to obtain standardized MRSA-infected chronic wounds in a diabetic mouse model that mimics human DFI pathophysiology. Not only did mice reveal signs of T1DM, such as hyperglycemia, polydipsia and polyuria [11], but experimentally inflicted wounds in diabetic mice showed an effective infection by MRSA that triggered continuous necrosis and infiltration of immune cells. This arrested state of necrosis associated with an inflammatory microenvironment hindered healing progression, representing key hallmarks of wound chronicity [2, 4]. Ultimately, this model depicts critical features of the DFI microenvironment that previous animal models of infected wounds failed to validate, including: i) the ability of MRSA to penetrate and spread within the wound bed, rather than being restricted to the scab [12–15]; ii) minimizing primary skin contraction that naturally occurs in rodents, which allows a healing mechanism by re-epithelization [12, 14, 16, 17]; and iii) control over the causative agent and dose of infection, circumventing the lack of standardization observed in models of naturally infected wounds that depend on housing conditions, source of animal colonies and host skin microbiome [18].

All techniques herein employed were simple, easily executed and optimized in terms of timing and number of interventions to minimize animal morbidity and mortality, although they should be expected, specially at later timepoints of infection. Importantly, the behavior and mobility of mice were not significantly impacted by dressings, that remained in place and were not removed by mice, even though they were caged in group.

Overall, this model offers a simple and suitable approach for studying emerging technologies of topical application for the treatment of MRSA-infected chronic wounds, that are difficult to test in the available animal models. Ultimately, it can be adapted to different diabetic animal models or to other pathophysiological conditions.

## Limitations

Wound infection with a single bacterial specie constitutes a limitation of this study. It would be relevant to further apply this protocol using polymicrobial biofilms to evaluate the interspecies relationship on the wound healing process.

## Supplementary Information

**Additional file 1: Table S1.** Animal welfare scoresheet and humane endpoints.

## Data Availability

The data generated and analyzed during the current study are available from the corresponding author on reasonable request.

## Abbreviations

DFI: Diabetic foot infection
MRSA: Methicillin-resistant *Staphylococcus aureus*
Dpi: Days post-infection
T1DM: Type 1 diabetes mellitus
STZ: Streptozotocin
MSA: Mannitol salt agar
CFU: Colony forming units
H&E: Hematoxylin and Eosin
