# Supplementary figures and images for: An optimized mouse model of Staphylococcus aureus infected diabetic ulcers
Source: BMC Res Notes. 2022 Sep 7;15:293. doi: 10.1186/s13104-022-06170-5 (PMC9450231; doi:10.1186/s13104-022-06170-5)

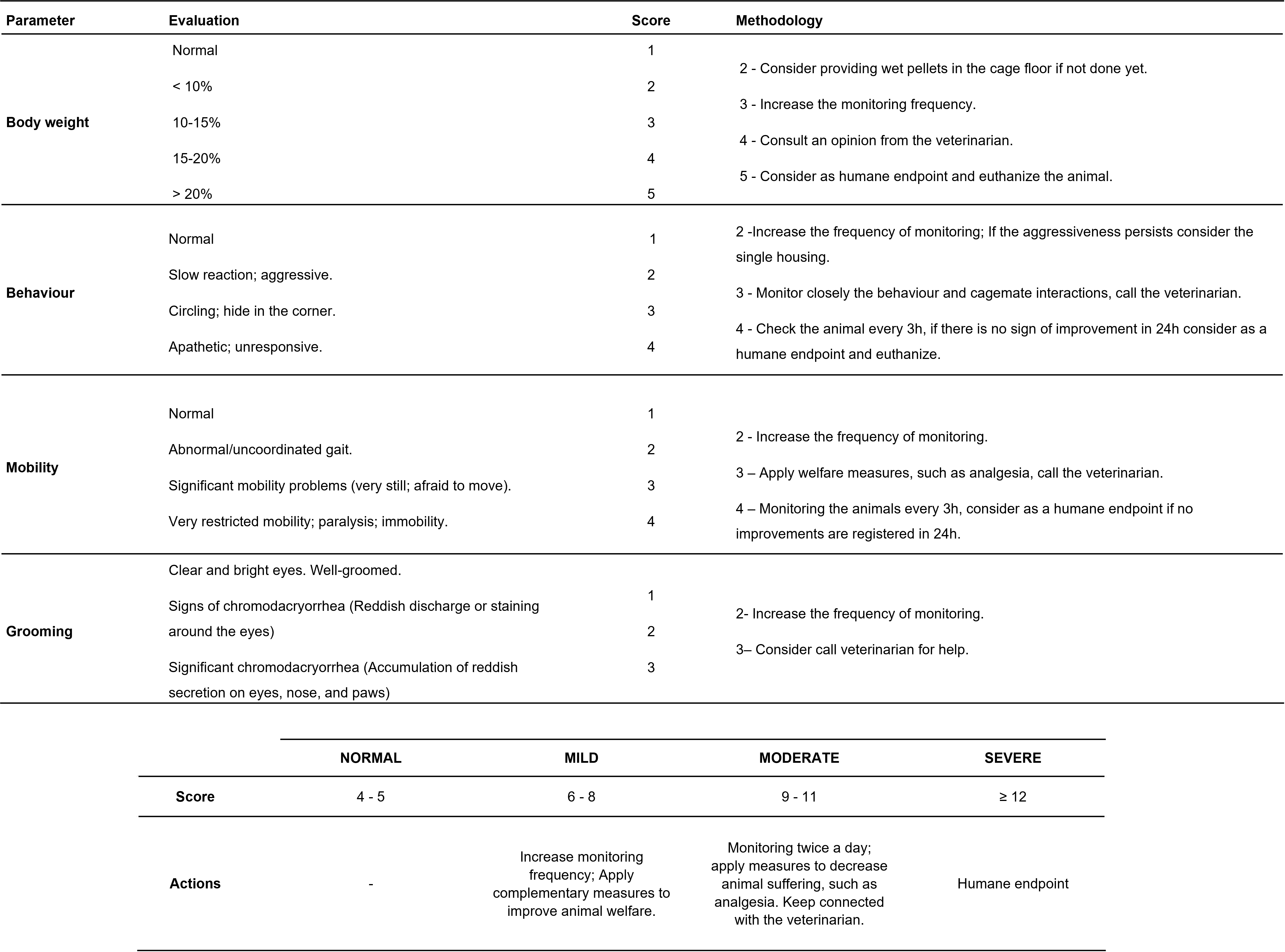

Supplement: Supplementary file 1 — Additional file 1: Table S1. Animal welfare scoresheet and humane endpoints. [file 13104_2022_6170_MOESM1_ESM.tif]
